# Implementation of Injectable Cabotegravir/Rilpivirine for Treatment of Human Immunodeficiency Virus in Patients With Substance Use Disorders at a Syringe Exchange Clinic

**DOI:** 10.1093/ofid/ofae640

**Published:** 2024-10-23

**Authors:** Amanda Perez, Shane Nieves, Jessica Meisner

**Affiliations:** Department of Medicine, University of Pennsylvania, Philadelphia, Pennsylvania, USA; Prevention Point Philadelphia, Philadelphia, Pennsylvania, USA; Prevention Point Philadelphia, Philadelphia, Pennsylvania, USA; Division of Infectious Diseases, Department of Medicine, University of Pennsylvania, Philadelphia, Pennsylvania, USA

**Keywords:** cabotegravir, human immunodeficiency virus, opioid use disorder, rilpivirine, syringe exchange

## Abstract

Cabotegravir + rilpivirine (CAB + RPV-LA) is a long-acting antiretroviral therapy (ART) that can be utilized for people with human immunodeficiency virus (HIV) who face barriers to daily ART. Here, we describe the implementation of a program that provides low-barrier access to CAB + RPV-LA for people with HIV and opioid use disorder at a syringe exchange.

## Graphical Abstract

**Graphical Abstract:**
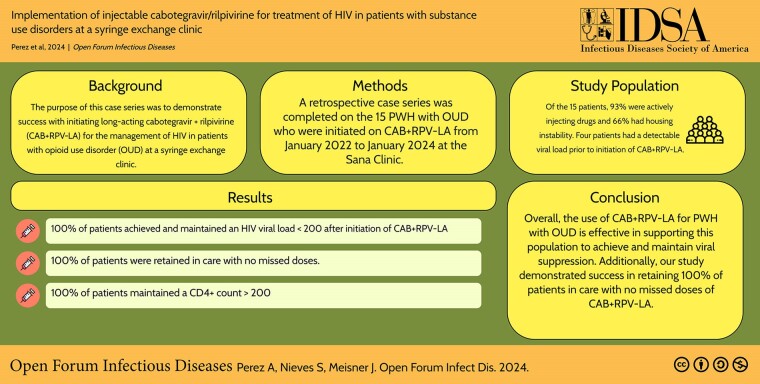
This graphical abstract is also available at Tidbit: https://tidbitapp.io/tidbits/implementation-of-injectable-cabotegravir-rilpivirine-for-treatment-of-hiv-in-patients-with-substance-use-disorders-at-a-syringe-exchange-clinic?utm_campaign=tidbitlinkshare&amp;utm_source=ITP

## Teaser

This case series looks at the two-year implementation of Injectable Cabotegravir+ Rilpivirine (CAB+RPV-LA) in a syringe exchange clinic with patients who have opioid use disorder and housing instability. We found 100% adherence and 100% suppression in all 15 patients.

An intramuscular formulation of cabotegravir (CAB), a human immunodeficiency virus (HIV) integrase strand transfer inhibitor, and rilpivirine (RPV), a nonnucleoside reverse transcriptase inhibitor, was the first long-acting (LA) regimen approved by the United States Food and Drug Administration for the treatment of HIV-1 in virologically suppressed adults. Clinical trials demonstrated that CAB + RPV-LA dosed every 8 weeks was noninferior for maintenance of viral suppression compared to oral therapy [1, 2].

Long-acting antiretroviral therapy (ART) has the potential to provide treatment for people with HIV (PWH) who face barriers to adherence or who are unable to achieve or maintain viral suppression on oral ART. One population that can benefit from long-acting treatment are PWH with opioid use disorder (OUD). Patients with OUD, and particularly those who inject drugs, have a higher rate of HIV acquisition and transmission [3].

Having HIV and substance use disorder is associated with delayed HIV care, decreased adherence to ART, and decreased rates of virologic suppression [4–6]. A significant association between heroin use and nonadherence to oral ART has also been demonstrated [7]. These data demonstrate the need for treatment options that can improve medication adherence in the HIV population.

To our knowledge, there is currently no literature demonstrating success of implementation of long-acting ART for HIV treatment in patients with OUD. The purpose of this case series is to demonstrate success with initiating CAB + RPV-LA for the management of HIV in patients with OUD at a syringe exchange clinic.

## METHODS

### Patient Consent Statement

This study was exempt from oversight and patient consent was not required by the Institutional Review Board at the Philadelphia Department of Public Health. All patient data were anonymized.

### Program Characteristics

The Sana Clinic at Prevention Point Philadelphia is a “one-stop shop” clinic that provides integrated HIV care, primary care, and medications for OUD for PWH with OUD. Prevention Point Philadelphia is a nonprofit harm reduction organization and syringe exchange program located in Kensington, Philadelphia. The physician at the Sana Clinic is an infectious disease and internal medicine specialist who has been caring for PWH for over a decade. Medical services are provided by appointment and on a walk-in basis twice a week.

The Sana clinic cares for 84 patients. All eligible patients were offered the option of starting CAB + RPV-LA. Eligibility criteria for CAB + RPV-LA were having no active hepatitis B, having no resistance to CAB or RPV, and having a viral load (VL) <20 000 copies/mL. Sana clinic partnered with a 340B pharmacy to obtain CAB + RPV-LA. Injections were covered by patients’ Medicaid insurance. Factors for switching to CAB +RPV-LA included having medications get lost or stolen, difficulty with adherence, and patient preference to not take a daily medication.

Initially, patients received 2 loading doses spaced 1 month apart. All subsequent injections were given every 2 months. A reminder card with the date of their next injection was provided at each visit. Phlebotomy limitations led to varying timelines between number of injections and time of follow-up laboratory tests. However, Sana clinic staff aimed to obtain follow-up labs and screening for sexually transmitted infections within 3 months of starting CAB + RPV-LA and every 3 months after.

All patients were assigned a medical case manager (MCM) who specialized in working with patients who inject drugs and who was located on-site at the syringe exchange. MCMs helped patient get identification cards, complete housing and food applications, and make appointments. MCMs called patients with phones and conducted street outreach if patients did not have a phone to remind them of appointments.

### Study Design and Participants

A retrospective case series was completed on the 15 PWH with OUD who were initiated on CAB + RPV-LA from January 2022 to January 2024 at the Sana Clinic. The electronic medical record (EMR) was reviewed to identify individuals aged ≥18 years with HIV and OUD who were initiated on CAB + RPV-LA during this period. These patients were started on CAB + RPV-LA on a rolling basis. The last patient initiated on CAB + RPV-LA was started 8 months before the end of the study period. The primary outcome was HIV viral suppression after initiation of CAB + RPV-LA. Secondary outcomes included retention/adherence rate, absolute CD4^+^ count, and adverse events. The decision to start on CAB + RPV-LA was based on shared decision-making with patients. Patients were given a $50 incentive after each loading dose and $100 for each subsequent dose.

### Data Collection

Variables extracted from the EMR were race, ethnicity, age, gender, sex, HIV VL before starting long-acting ART, last HIV VL on long-acting ART, CD4^+^ cell count prior to starting long-acting ART, CD4^+^ cell count after starting long-acting ART, CD4^+^ cell percentage, history of intravenous drug use (IVDU), nonopioid drug use, hepatitis B serologies, hepatitis C antibody status, housing status, prior ART medications, missed CAB + RPV-LA doses, late doses, and mortality. The data gathered were securely stored on an internal server.

### Data Analysis

We used descriptive statistics to summarize the demographics, clinical characteristics, and outcomes of the study population. The absolute and relative frequencies (%) were calculated for all variables.

## RESULTS

### Patient Characteristics

A total of 15 patients (3 cisgender women and 12 cisgender men) were included. Three (20%) patients were of Hispanic/Latinx ethnicity. Two (13.3%) were African American. Additionally, 14 (93%) had active IVDU, and 10 (66.6%) had unstable living conditions. All patients had previously been on oral ART with varying adherence rates. Other demographic characteristics and comorbidities are shown in Table 1.

**Table 1.** Demographic Information for Patients (N = 15) Started on Long-Acting Cabotegravir + Rilpivirine at the Sana Clinic

Characteristic	No. (%)
Race	
African American	2 (13.3)
White	12 (80.0)
Asian	0 (0.0)
Other	1 (6.7)
Ethnicity	
Hispanic/Latinx	3 (20.0)
Not Hispanic/Latinx	12 (80.0)
Age, y	
18–29	1 (6.7)
30–39	6 (40.0)
40–49	5 (33.3)
≥50	3 (20.0)
Gender	
Male	12 (80.0)
Female	3 (20.0)
Transgender	0 (0.0)
Sex	
Male	12 (80.0)
Female	3 (20.0)
Other/not disclosed	0 (0.0)
IVDU in last year/active IVDU	
Yes	14 (93.3)
No	1 (6.7)
Non-opioid use	
Marijuana	0 (0.0)
Methamphetamines	1 (6.7)
Cocaine	5 (33.3)
Other	1 (6.7)
Multiple	4 (26.7)
NA	4 (26.7)
Hepatitis B serologies	
Immune (natural/cAb^+^)	5 (33.3)
Nonimmune	0 (0.0)
Immune (vaccinated)	10 (66.7)
Hepatitis C serologies	
Ab^+^	14 (93.3)
Ab^−^	1 (6.7)
Housing status	
Street homeless	9 (60.0)
Shelter	1 (6.7)
Housed	5 (33.3)
ART medication prior to CAB + RPV-LA	
BIC/FTC/TAF	13 (86.7)
EVG/COBI/FTC/TAF	2 (13.3)
Other	0 (0.0)
Missed doses since start	
Yes	0 (0.0)
No	15 (100.0)
Dose >2 wk late	
Yes	1 (6.7)
No	14 (93.3)
Mortality	
No	14 (93.3)
Yes	1 (6.7)

Abbreviations: Ab^+^, antibody positive; Ab^–^, antibody negative; ART, antiretroviral therapy; BIC, bictegravir; cAb^+^, core antibody positive; CAB + RPV-LA, long-acting cabotegravir + rilpivirine; COBI, cobicistat; EVG, elvitegravir; FTC, emtricitabine; IVDU, intravenous drug user; NA, not applicable; TAF, tenofovir alafenamide.

### Primary/Secondary Outcomes

Patients’ VL and CD4^+^ cell count were checked just prior to starting CAB + RPV-LA. Prior to initiation, 11 (73.3%) patients had an HIV VL <200 copies/mL and 4 (26.7%) had a VL >200 copies/mL. No patients had a CD4 count <200 cells/μL.

The primary outcome of the study was virologic suppression. All of the patients initiated on CAB + RPV-LA successfully achieved HIV viral suppression on follow-up laboratory testing (Table 1). Viral suppression was achieved in all patients not previously suppressed on the first set of follow-up labs.

Secondary outcomes were retention/adherence rate, CD4^+^ count, and adverse events. There was a 100% retention rate among patients started on CAB + RPV-LA and no missed doses during the study period. However, 1 patient received 1 dose that was >2 weeks late (Table 2). Missed doses were defined as being >4 weeks late, which is when patients would need to be reloaded with 2 monthly injections. Anything <4 weeks was defined as a late dose. Additionally, 100% of patients maintained a CD4^+^ count >200 cells/μL.

**Table 2.** Outcomes for People With Human Immunodeficiency Virus With Opioid Use Disorder After Initiation of Long-Acting Cabotegravir + Rilpivirine at the Sana Clinic

Outcome	Prior to Starting LA ART	Final Collection After Starting LA ART
HIV RNA, copies/mL		
0–200	11 (73.3)	15 (100.0)
201–1000	2 (13.3)	0 (0.0)
1001–5000	2 (13.3)	0 (0.0)
5001–10 000	0 (0.0)	0 (0.0)
>10 000	0 (0.0)	0 (0.0)
CD4 count, cells/μL		
<200	0 (0.0)	0 (0.0)
200–400	2 (13.3)	1 (6.7)
400–600	4 (26.7)	5 (33.3)
>600	9 (60.0)	9 (60.0)
CD4 percentage		
<15%	1 (6.7)	0 (0.0)
15%–25%	3 (20.0)	4 (26.7)
>25%	11 (73.3)	11 (73.3)

Data are presented as No. (%).

Abbreviations: ART, antiretroviral therapy; HIV, human immunodeficiency virus; LA, long-acting.

In terms of adverse outcomes, 1 patient died during the study period due to an opioid overdose after the patient resumed engaging in substance use. This patient was adherent to CAB + RPV-LA until death. This death was unrelated to administration of CAB + RPV-LA.

## DISCUSSION

To our knowledge, this is the only study describing the successful implementation of long-acting ART for HIV treatment in patients with OUD at a syringe exchange clinic. However, some studies have demonstrated success in other vulnerable populations who face similar social inequities as PWH with OUD.

PWH who use drugs are more than twice as likely to face social inequities, such as housing instability. Living outdoors or in temporary housing is associated with lower rates of virologic suppression compared to being stably housed [8]. A recent study demonstrated effectiveness of using CAB + RPV-LA with and without viral suppression in an urban clinic that served patients with housing instability and stimulant use. In this study, patients without viral suppression who began CAB + RPV-LA were able to achieve viral suppression or have a 2-log decline in VL within 1 month of the first injection [9]. Like our sample, Christopoulos et al demonstrated an 100% adherence rate to CAB + RPV-LA [9]. This study demonstrated the potential for success of long-acting ART in vulnerable populations with barriers to daily medication adherence and those who were not previously virologically suppressed.

Our 100% adherence rate is notable as previous studies have demonstrated that PWH and OUD have decreased retention in care compared to individuals without OUD [10]. Our findings support that embedding clinics that provide access to long-acting ART in syringe services programs, or other social services frequented by PWH with OUD, can help to overcome barriers with adherence.

There are several limitations to this study. First, our sample size was limited to a small number of patients at 1 clinic in Philadelphia. Most of the patients were cisgender men, which may limit generalizability to women or other genders. Selection bias is another limitation of the study as patients opted in to receiving CAB + RPV-LA. Additionally, patients who received CAB + RPV-LA were provided monetary incentives. However, patients on oral ART at the Sana clinic are also provided incentives. Therefore, it is unclear how incentives influenced patients’ rate of adherence to CAB + RPV-LA.

Overall, the use of CAB + RPV-LA for PWH with OUD is effective in supporting this population to achieve and maintain viral suppression. Since this study was conducted, an additional 15 patients were initiated on CAB + RPV-LA and the program continues to have a 100% retention rate aside from 2 patients whose care was transferred due to incarceration. There is a need for hybrid implementation-effective trials and further studies with larger cohort sizes and more diverse samples to support success of CAB + RPV-LA in PWH with OUD.

## Notes

***Acknowledgments.*** The authors thank Prevention Point Philadelphia and the Sana Clinic staff for their excellent care management and support. We are also grateful to the clinic's medical case management team who were instrumental to the implementation of this program.

***Author contributions.*** A. P.: Methodology, data collection and analysis, writing–original draft, reviewing and editing. S. N.: Data collection and analysis, writing–reviewing and editing. J. M.: Conceptualization, methodology, data collection and analysis, writing–original draft, reviewing and editing.
